# Pressure Dependence of ^15^N Chemical Shifts in Model Peptides Ac-Gly-Gly-X-Ala-NH_2_

**DOI:** 10.3390/ma5101774

**Published:** 2012-09-27

**Authors:** Joerg Koehler, Markus Beck Erlach, Edson Crusca, Werner Kremer, Claudia E. Munte, Hans Robert Kalbitzer

**Affiliations:** 1 Institute of Biophysics and Physical Biochemistry and Centre of Magnetic Resonance in Chemistry and Biomedicine, University of Regensburg, Regensburg 93040, Germany; E-Mails: joerg.koehler@biologie.uni-regensburg.de (J.K.); markus.beck-erlach@biologie.uni-regensburg.de (M.B.E.); werner.kremer@biologie.uni-regensburg.de (W.K.); 2 Physics Institute of São Carlos, University of São Paulo, São Carlos 13566-590, Brazil; E-Mails: edsoncrusca@yahoo.com.br (E.C.); claudia.munte@ifsc.usp.br (C.E.M.)

**Keywords:** tetrapeptide, high pressure, NMR spectroscopy, random-coil, chemical shift, J-coupling, nitrogen, amide group, backbone

## Abstract

High pressure NMR spectroscopy has developed into an important tool for studying conformational equilibria of proteins in solution. We have studied the amide proton and nitrogen chemical shifts of the 20 canonical amino acids X in the random-coil model peptide Ac-Gly-Gly-X-Ala-NH_2_, in a pressure range from 0.1 to 200 MPa, at a proton resonance frequency of 800 MHz. The obtained data allowed the determination of first and second order pressure coefficients with high accuracy at 283 K and pH 6.7. The mean first and second order pressure coefficients <B_1_^15N^> and <B_2_^15N^> for nitrogen are 2.91 ppm/GPa and −2.32 ppm/GPa^2^, respectively. The corresponding values <B_1_^1H^> and <B_2_^1H^> for the amide protons are 0.52 ppm/GPa and −0.41 ppm/GPa^2^. Residual dependent ^1^J_1H15N_-coupling constants are shown.

## 1. Introduction

High pressure nuclear magnetic resonance spectroscopy (HP-NMR) is a powerful tool for investigating thermodynamic behavior of macromolecules in solution at atomic resolution (for a review see e.g., [1]). Application of pressure has direct effects on molecular properties such as bond lengths in hydrogen bonds but more importantly usually shifts conformational equilibria in the structural ensemble of biological macromolecules such as proteins by its contribution to the free energies of the system. These structural changes influence all NMR parameters but notably chemical shifts are very precisely observable. Since proteins have a highly anisotropic compressibility the observed chemical shift changes show large variations from atom to atom. In rigid proteins such as the basic pancreatic trypsin inhibitor (BPTI), the pressure-induced changes of chemical shifts can be fitted well by a linear dependence for almost all atoms [2]. In contrast, in proteins that exist in more than one conformational state in solution, usually non-linear pressure responses are found. The first example was the histidine containing protein (HPr) from *S. carnosus* that shows strong deviations from the linearity in the pressure response. Usually, these shifts are observed in regions of the protein where interactions with small ligands or other proteins occur. In HPr it is the active-centre loop around His15 (interaction site with enzyme I and enzyme II) and in the regulatory helix B (interaction site with the HPr-kinase/phosphatase) [3]. These regions should also show a higher internal mobility [4] and compressibility since structural transitions should be possible.

At a given temperature *T_0_* and pressure *p* the chemical shift δ*^k^* of a given nucleus *k* in a multistate system depends on the exchange correlation times *τ_ij_^k^* and the chemical shift differences *Δω_ij_^k^* of the states *i* and *j*. In many cases one can assume that fast exchange prevails for all states *i* and *j* with

(1) $$|\Delta \omega_{ij}^{k}\tau_{ij}^{k}|\;\;\;\;<<1$$

The fast exchange condition should also hold for the substates assigned to an expectation value <δ*_ι_^k^*> of a state *i*.

The pressure dependence of the chemical δ*^k^* is than given by

(2) $$\delta^{k}(T_{0},p)=\frac{1}{Z}\sum_{i=1}^{N}<\delta_{i}^{k}>{p^{\prime }}_{i}=\frac{1}{Z}\sum_{i=1}^{N}<\delta_{i}^{k}>\exp\left(-\frac{1}{RT}\left(G_{i}^{0}+V_{i}^{0}(p-p_{0})+\frac{1}{2}\beta_{i}^{0}{\left(p-p_{0}\right)}^{2}\right)\right)$$

with *p'_i_* the population of state *i*, *Z* the states sum, *G_i_^0^* the free energy at (*p_0_*, *T_0_*), *V_i_^0^* the partial molar volume at (*p_0_*, *T_0_*), *β_i_^0^* the compressibility factor at (*p_0_*, *T_0_*) and *R* the gas constant. For the interpretation of the data it is important to separate trivial chemical shift changes as they occur also in unfolded peptides from structurally or functionally important pressure induced changes. The simplest way to do such a correction is just subtracting the relatively small pressure dependent chemical shifts of model peptides from the experimental data. The small pressure dependent changes of the chemical shift *δ (T, p)* can be fitted sufficiently well by a second order Taylor expansion as

(3) $$\delta (T_{0},p)=\delta_{0}(T_{0},p_{0})+B_{1}\left(p-p_{0}\right)+B_{2}{\left(p-p_{0}\right)}^{2}$$

with *T* the temperature, *p* the pressure and *B_1_* and *B_2_* the first and second order pressure coefficients, respectively [5].

Such a correction has been applied earlier to the pressure response of the human prion protein [6] and helped to identify stretches with residual structures in the disordered N-terminal tail of the protein. However, only ^1^H pressure coefficients were reported by Arnold *et al.* [5]. In the present study we will report ^15^N shifts and refined ^1^H chemical shifts for the amide groups of the 20 canonical amino acids X in the tetrapeptide Ac-Gly-Gly-X-Ala-NH_2_. The N- and C-terminally unprotected tetrapeptide Gly-Gly-X-Ala has been introduced as random coil model by Wüthrich and Bundi [7].

## 2. Results and Discussion

### 2.1. Pressure Dependence of the Backbone ^15^N Chemical Shifts

**Figure 1:**
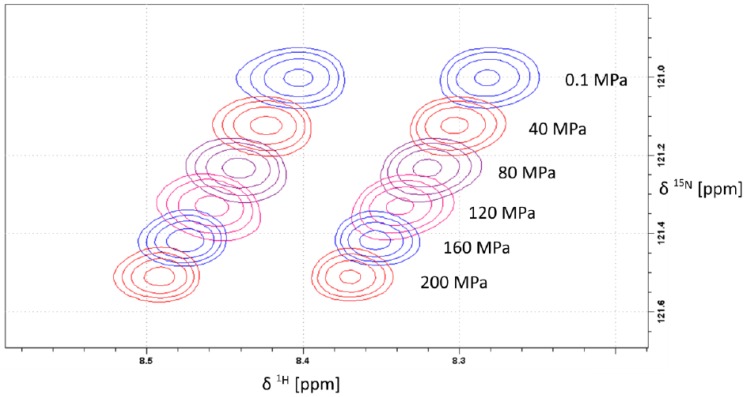
Pressure dependent ^1^H-^15^N-HSQC spectra of backbone amide region of arginine. The samples contained 5 mM Ac-Gly-Gly-Arg-Ala-NH_2_ in 20 mM Tris-HCl in 90% H_2_O/10% D_2_O, pH 6.7. Measurements were performed at 283 K. Data processing was done with Gaussian filter in both directions using negative line broadenings of −10 Hz and −3 Hz for ^15^N and ^1^H, respectively.

Generally, the largest pressure dependent shift changes in protein backbones are expected for the amide groups since they are involved in hydrogen bonding. Figure 1 shows a typical pressure dependence of amide chemical shifts in an ^1^H–^15^N HSQC spectrum. Since the amino acid X in the tetrapeptide was enriched in ^15^N as well as ^13^C, ^1^H and ^13^C decoupling was performed in the indirect dimension. In the direct dimension broadband decoupling was not possible because the long acquisition time was required for the necessary resolution. All ^15^N resonances shift downfield with increasing pressure, most probably because of the expected shortening of the hydrogen bonds with pressure [8]. Such an effect has been described earlier by Kalbitzer *et al.* [3].

In first approximation the pressure dependence of backbone ^15^N resonances is linear with a mean first order pressure coefficient *<B_1_^15N^>* value of 2.91 ppm/GPa (Table 1). Only small deviations from the linearity are observed, corresponding to a mean second order pressure coefficient *<B_2_^15N^>* of −2.32 ppm/GPa^2^. 

**Table 1:** Pressure dependence of backbone ^15^N chemical shifts. The samples contained 5 mM Ac-Gly-Gly-X-Ala-NH_2_ in 20 mM Tris-HCl in 90% H_2_O/10% D_2_O, pH 6.7. HSQC measurements were performed at 283 K in a pressure range from 0.1 MPa to 200 MPa. *δ_0_* corresponds to chemical shift at atmospheric pressure, *B_1_* and *B_2_* were obtained by fitting the pressure dependence of the chemical shift *δ* to Equation (3). The errors given are standard deviations from the fitting algorithm. The errors of the *δ_0_* and ^1^J_1H15N_ can be estimated as 0.01 ppm and 0.1 Hz, respectively.

	*δ_0_*^15N^	*B_1_*^15N^			*B_2_*^15N^			^1^J_1H15N_
	(ppm)	(ppm/GPa)			(ppm/GPa^2^)			(Hz)
**Ala**	124.39	2.74	±	0.03	−1.49	±	0.16	−97.5
**Arg**	121.00	3.01	±	0.04	−2.44	±	0.21	−95.5
**Asn**	119.12	2.98	±	0.17	−1.73	±	0.82	−95.9
**Asp**	120.94	2.94	±	0.07	−2.58	±	0.36	−96.0
**Cys**	119.21	2.75	±	0.08	−1.87	±	0.41	−96.0
**Gln**	120.25	2.87	±	0.06	−2.15	±	0.29	−93.5
**Glu**	120.95	2.78	±	0.17	−1.66	±	0.83	−99.5
**Gly**	109.22	3.79	±	0.08	−2.16	±	0.37	−96.9
**His pH 4.0**	118.34	3.42	±	0.08	−2.86	±	0.38	−98.2
His pH 8.5*	120.33	3.15	±	0.10	−3.53	±	0.50	–
**Ile**	120.39	2.35	±	0.03	−1.89	±	0.17	−92.9
**Leu**	122.07	2.65	±	0.10	−2.92	±	0.48	−96.2
**Lys**	121.29	3.00	±	0.03	−2.24	±	0.13	−94.7
**Met**	120.17	2.76	±	0.05	−1.89	±	0.22	−92.7
**Phe**	120.62	3.01	±	0.04	−3.65	±	0.19	−95.5
Pro trans*	134.78	2.45	±	0.02	−1.76	±	0.10	–
Pro cis*	135.03	1.36	±	0.02	−0.38	±	0.10	–
**Ser**	116.27	3.41	±	0.15	−2.46	±	0.70	−95.7
**Thr**	114.14	3.28	±	0.05	−2.36	±	0.24	−93.8
**Trp**	121.43	3.35	±	0.03	−2.90	±	0.17	−95.5
**Tyr**	120.33	3.56	±	0.07	−4.52	±	0.32	−93.9
**Val**	119.41	2.32	±	0.02	−1.70	±	0.12	−95.0

Note: * Data acquisition performed via HA(CA)N experiment.

**Figure 2:**
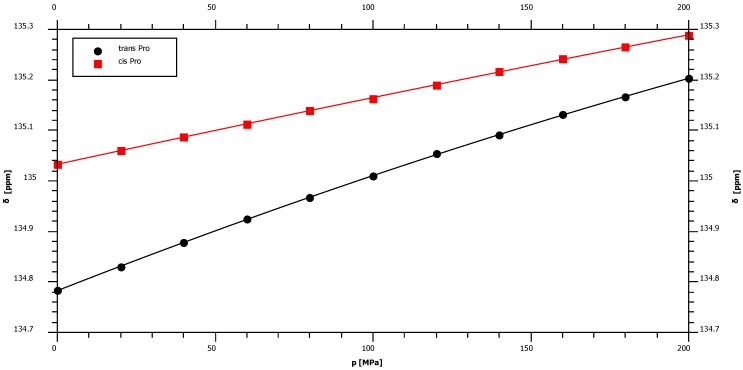
Pressure dependence of backbone ^15^N chemical shift resonances of proline. The samples contained 5 mM Ac-Gly-Gly-Pro-Ala-NH_2_ in 20 mM Tris-HCl in 90% H_2_O/10% D_2_O, pH 6.7. Measurements were performed at 283 K. The chemical shift *δ* was fitted as a function of pressure *p* to Equation (3) with the parameters given in Table 1.

**Figure 3:**
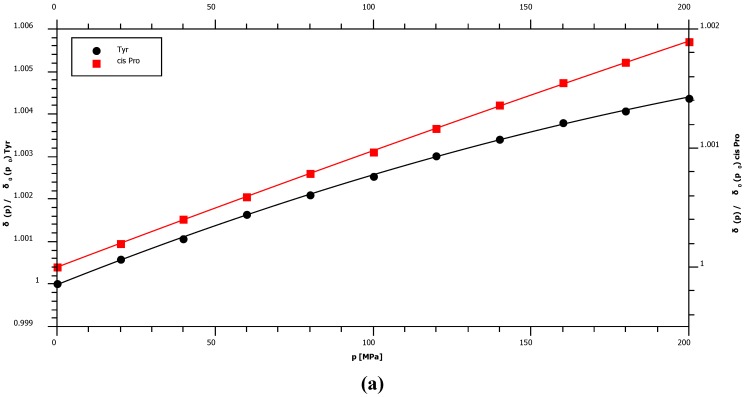

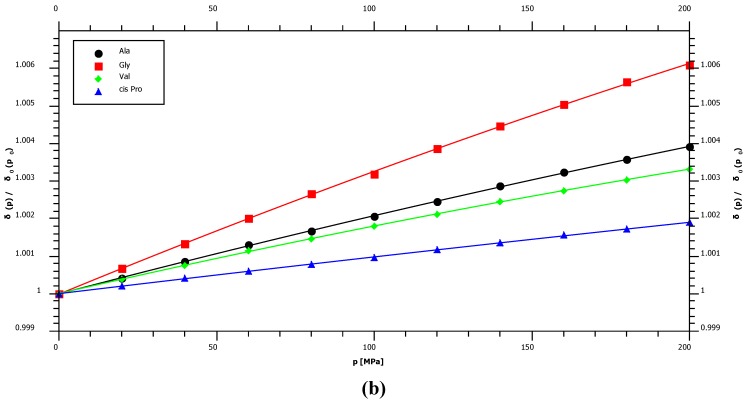
Pressure dependent ^15^N chemical shift changes of selected residues. The chemical shifts *δ* are plotted as a function of pressure *p* for some residues X. For a better comparison the inverse of the initial value at ambient pressure was used as normalization factor for each data series. (**a**) Non-linearity of the pressure dependence of cis-proline and tyrosine residues. (**b**) The pressure dependence of chemical shifts of selected residues.

With 1.36 ppm/GPa cis-proline shows the smallest ^15^N first order pressure response of all amino acids but an average pressure response in the trans-configuration (Figure 2, Table 1). This is in agreement with the supposed hydrogen bonding mechanism that should be negligible in the cis-configuration since the water interaction with peptide nitrogen is largely blocked. The largest pressure induced ^15^N chemical shift changes are observed for glycine residues with a first order pressure coefficient of 3.79 ppm/GPa (Table 1). Concerning the second order coefficients again cis-proline shows the smallest non-linearity of the pressure dependent ^15^N chemical shifts (−0.38 ppm/GPa^2^) and tyrosine the largest one (−4.52 ppm/GPa^2^) (Table 1). Figure 3 shows that these differences in non-linearity are clearly significant. For nitrogen backbone resonances all *B_2_* values are negative suggesting an asymptotic behavior at very high pressures.

**Figure 4:**
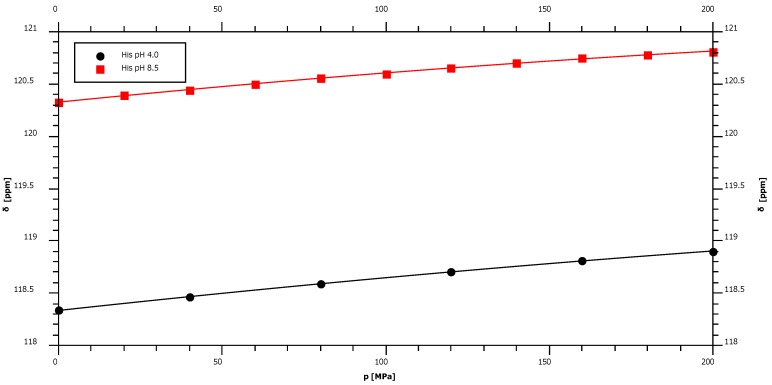
Pressure dependence of backbone ^15^N chemical shift resonances of histidine Ac-Gly-Gly-His-Ala-NH_2_. Sample composition, experimental conditions and data fitting are described in Table 1. The sample was measured at pH 4.0 and pH 8.5.

In random coil peptides histidine residues are in equilibrium between protonated and deprotonated states of the imidazole ring. In Gly-Gly-His-Ala the pK_a_ is 7.0 [7]. The protonation/deprotonation equilibrium of the ring may also influence the pressure induced shifts at the backbone atoms. Therefore, the pressure dependence of the nitrogen chemical shift was measured at two different pH-values, at pH 4.0 where the ring should be fully protonated and pH 8.5 where the ring is fully deprotonated. Since at high pH the amide proton exchange is too fast for a polarization transfer from the amide proton to the nitrogen, an HA(CA)N experiment was performed where the primary polarization transfer occurs from the H^α^-proton. The backbone amide resonance is shifted upfield at lower pH and the first order pressure coefficient increases slightly (Figure 4, Table 1).

### 2.2. Measurement of the One-Bond Amide Coupling Constants

In our tetrapeptides a residue dependent variation of the ^1^J_1H15N_-coupling constant of nearly 7 Hz is shown in Table 1. Significant changes of the ^1^J_1H15N_-coupling constant were observed in HPr from *S. carnosus* with a pressure dependent increase of the coupling constant up to 20 Hz [3].

### 2.3. Pressure Dependence of the Amide ^1^H Chemical Shifts

Pressure dependent chemical shifts were measured earlier for Gly-Gly-X-Ala by Arnold *et al.* [5] at a proton resonance frequency of 500 MHz in the pressure range from 0.1 to 200 MPa. They were determined in 50 mM phosphate buffer at pH 5.0 and 5.4 at 305 K. Since our data were recorded at different experimental conditions (20 mM Tris-HCl, pH 6.7, 283 K) it seems to be worthwhile to compare them with the older data for obtaining information about the pH and temperature effects. With Ac-Gly-Gly-X-Ala-NH_2_ we also used an N-terminally and C-terminally protected peptide where the N- and C-terminus remain uncharged independent of the pH used. In addition they were measured at 800 MHz and thus promise a higher accuracy of the obtained pressure coefficients (Table 2). The lower temperature of 283 K used in our study is expected to lead to a mean downfield shift of 0.17 ppm [9] of the amide resonance frequencies relative to the values reported by Arnold *et al.* [5] for 305 K. In contrast to this expectation only a very small shift deviation could be observed (0.005 ± 0.05 ppm). This is probably due to the protection of the N- and C-terminal charged groups. In addition a pH-dependent upfield shift might partially compensate the temperature dependent effect for some residues [5]. The chemical shift for histidine was measured at pH 4.0 (Table 2) since at high pH the amide proton exhanges too fast with the bulk water to be observable. Since the pK of histidine is 7.0 in the tetrapeptide [7] the value given here corresponds to a positively charged, fully protonated imidazole side chain. At neutral pH one would expect an upfield shift of the amide proton resonance and a downfield shift of the nitrogen resonance [9]. The pH dependence of the histidine nitrogen chemical shifts reported here are in aggreement to the expectation (Table 1).

**Table 2:** Pressure dependence of backbone amide H^N^ chemical shifts. The samples contained 5 mM Ac-Gly-Gly-X-Ala-NH_2_ in 20 mM Tris-HCl in 90% H_2_O/10% D_2_O, pH 6.7. Measurements were performed at 283 K in a pressure range from 0.1 MPa to 200 MPa. *δ_0_* corresponds to chemical shift at atmospheric pressure, *B_1_* and *B_2_* were obtained by fitting the pressure dependence of the chemical shift *δ* to Equation (3). The errors given are standard deviations from the fitting algorithm. The error of the *δ_0_* can be estimated as 0.001 ppm.

	*δ_0_*^HN^	*B_1_*^HN^			*B_2_*^HN^		
	(ppm)	(ppm/GPa)			(ppm/GPa^2^)		
**Ala**	8.368	0.45	±	0.02	−0.15	±	0.08
**Arg**	8.342	0.48	±	0.02	−0.22	±	0.10
**Asn**	8.503	0.50	±	0.03	−0.42	±	0.14
**Asp**	8.433	0.56	±	0.02	−0.48	±	0.09
**Cys**	8.418	0.55	±	0.01	−0.49	±	0.05
**Gln**	8.433	0.44	±	0.03	−0.17	±	0.13
**Glu**	8.539	0.25	±	0.02	−0.03	±	0.08
**Gly**	8.449	0.24	±	0.01	−0.10	±	0.06
**His pH 4.0**	8.484	0.51	±	0.01	−0.47	±	0.06
**Ile**	8.154	0.64	±	0.03	−0.64	±	0.14
**Leu**	8.279	0.52	±	0.02	−0.50	±	0.12
**Lys**	8.317	0.55	±	0.02	−0.49	±	0.10
**Met**	8.398	0.49	±	0.02	−0.31	±	0.10
**Phe**	8.263	0.55	±	0.02	−0.68	±	0.10
**Ser**	8.434	0.64	±	0.05	−0.65	±	0.26
**Thr**	8.269	0.75	±	0.02	−0.56	±	0.11
**Trp**	8.129	0.58	±	0.02	−0.42	±	0.11
**Tyr**	8.190	0.67	±	0.05	−0.82	±	0.25
**Val**	8.232	0.58	±	0.02	−0.21	±	0.08

**Figure 5:**
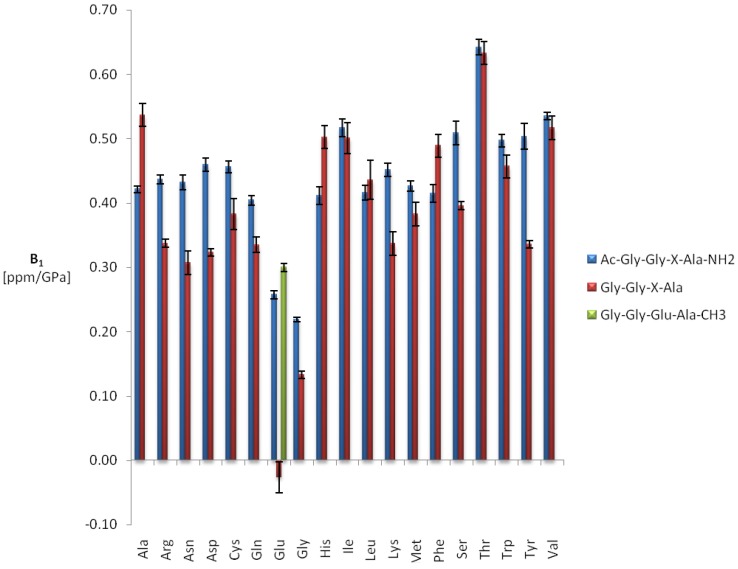
First order amide proton pressure coefficients in Ac-Gly-Gly-X-Ala-NH_2_ and Gly-Gly-X-Ala. Sample composition, experimental conditions and data fitting for Ac-Gly-Gly-X-Ala-NH_2_ (blue) are described in Table 2. The values for Gly-Gly-X-Ala (red) were taken from Arnold *et al.* [5] and were recorded for 5 mM tetrapeptide in 50 mM phosphate buffer, pH 5.4 at 305 K. The glutamate pressure coefficients of Gly-Gly-Glu-Ala-CH_3_ (green) [10] were recorded under the same experimental conditions as those of Gly.Gly-Glu-Ala.

The average first and second order shift coefficients *<B_1_^1H^>* and *<B_2_^1H^>* are 0.52 ppm/GPa and −0.41 ppm/GPa^2^ in Ac-Gly-Gly-X-Ala-NH_2_. In Gly-Gly-X-Ala they are with 0.42 ppm/GPa and −0.18 ppm/GPa^2^ somewhat smaller. The first order pressure coefficients are positive in both peptides, that is a downfield shift of the ^1^H amide resonances with pressure is observed. The only exception described by Arnold *et al.* [5] is glutamate where a negative first order pressure coefficient and a large second order coefficient had been observed. However, these unusual pressure coefficients are due to an interaction with the negative charge of the C-terminal carboxyl group since its methylation [10] or amination leads to a “normal” behavior of the amide resonance (Figure 5).

For the first order pressure coefficients the largest differences between the two peptides are observed for glutamate (0.28 ppm/GPa), followed by tyrosine and asparagine with 0.17 ppm/GPa and 0.14 ppm/GPa, respectively. As already observed for the amide nitrogen resonances the second order coefficients for protons are negative for all amino acids leading to a maximum in the pressure dependence at high pressures but probably are only an approximation for an asymptotic behavior at high pressures. In the data published by Arnold *et al.* [5] the sign of the second order pressure coefficients vary from amino acid to amino acid. This is most probably due to the lower NMR frequency used in the earlier study and thus a potentially larger error of measurements.

### 2.4. pH-Dependence of the Pressure Response

Amide proton shifts in random-coil peptides can be influenced by temperature and pH (see e.g., [9,11]). At ambient pressure the temperature dependence of the amide chemical shifts can be predicted sufficiently well from the amino acid specific temperature coefficients [9]. However, since the pK values of charged groups of the buffer as well as of the peptides are pressure dependent, pressure may induce a pH shift in the solution that also may influence the observed chemical shifts. Tris-HCl shows only a relatively small change of its pK-value with pressure compared to other buffers such as phosphate buffers [12]. This is the reason why it is often used as buffer in high-pressure NMR experiments on proteins. For having similar conditions we added Tris-HCl also for the study of the model peptides, although its buffer capacity is very low at pH 6.7. Here it can be used as pH-probe since the chemical shift dependence of the Tris-signal on pressure was studied in detail by Huberth [13]. At 200 MPa the pH increases in average by 0.01 pH units. The study by Arnold *et al.* [5] showed that the pressure coefficients of model peptides are only significantly influenced by pH when the side chain pK value is close to pH-value of the experiments. This is not the case in our study, only the pK-value of histidine is close enough to pH 6.7 but here the experiments were performed in the fully protonated and deprotonated state at pH 4.0 and 8.5 (Table 1).

## 3. Experimental Section

### 3.1. Synthesis of Peptides

Uniformly ^15^N and ^13^C enriched and N-terminally Fmoc protected amino acids were purchased from Sigma Aldrich (St. Louis, MO, USA) with an isotope enrichment larger than 98%. Side chains were protected by Pbf (Arg), Trt (Asn, Gln, His), Boc (Lys, Trp), tBu (Ser, Thr, Tyr), and OtBu (Asp, Glu). Solvents and reagents were obtained from Merck (Darmstadt, Germany). The tetrapeptides Ac-Gly-Gly-X-Ala-NH_2_ were synthesized by solid phase synthesis using a Rink Amide resin and Fmoc (N-(9-fluorenyl)methoxycarbonyl) N-terminal group protection of amino acids. Coupling of all amino acids was achieved using three equivalents of HBTU (O-Benzotriazole-N.N.N’.N’-tetramethyl-uronium-hexafluoro-phosphate) and two equivalents of DIEA (N.N-diisopropylethylamine) for each equivalent of Fmoc amino acid. When the peptide chain was finished, a mixture of 10% acetic anhydride in DMF (dimethylformamide) was used for N-terminal acetylation. The peptides were cleaved from the resin and the side-chain protecting groups were removed simultaneously with appropriate TFA/scavengers cocktails. The identity and purity of all peptides was confirmed by ESI-MS (Bruker, Billerica, MA, USA) and RP-HPLC (Waters, Milford, MA, USA).

### 3.2. Sample Preparation

The peptide concentration was 5 mM in aqueous solution of 90% H_2_0 and 10% D_2_O. Additionally 20 mM perdeuterated Tris-HCl (tris(hydroxymethyl)aminomethane hydrochloride) and 0.5 mM DSS (4.4-dimethyl-4-silapentane-sulfonic acid) were added. In general, the pH value was adjusted to 6.7 with a Hamilton Spintrode attached to a Beckman Coulter pH-meter. Only histidine was measured at pH 4.0 and pH 8.5. The measured pH-values have not been corrected for the deuterium isotope effect.

### 3.3. High Pressure System

**Figure 6:**
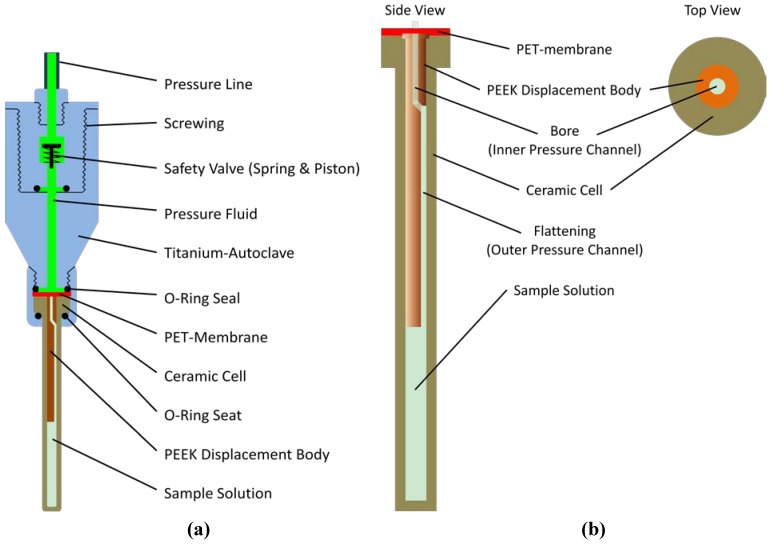
High pressure autoclave. (**a**) The high pressure autoclave holding the ceramic cell is similar to that provided by Daedalus Corporation [14], but has additionally a safety valve analogous to that described earlier by Beck Erlach *et al.* [15]; (**b**) A PET membrane was posed on top of the ceramic cell, and a bored and flattened cylindrical displacement body was inserted into the tube.

A homebuilt online-pressure system according to Yamada-method [16] was used. Pressure produced by a homemade manually operated piston compressor was transmitted via a high pressure line (High Pressure Equipment Company, Linden, PA, USA) by methylcyclohexane to the high pressure ceramic cell (with an outer diameter of 5 mm and an inner diameter of 3 mm) from Daedalus Innovations LLC (Aston. PA. USA). A PET (polyethylene terephthalate) membrane acts as a flexible separator between the pressure fluid and the aqueous sample. To reduce the volume of the ceramic cell a cylindric PEEK (polyether-ether-ketone) displacement body was used. A full-metal autoclave connects the ceramic cell with the closed pressure line (Figure 6). It is similar to the original autoclave provided by Daedalus Innovations [14] but is produced from titanium and contains a safety valve [15] that closes rapidly when the cell brakes.

### 3.4. NMR Spectroscopy

All NMR experiments were carried out on an 800 MHz BRUKER Avance spectrometer with a QXI probe at 283 K and the magnetic field was locked to D_2_O. Temperature calibration was done before each sample-change via the difference of the resonance lines of the hydroxyl- and methyl-group protons in 100% methanol according to [16]. ^15^N resonances of amide-groups were detected indirectly via ^1^H–^15^N-HSQC (Hetero-Single-Quantum-Coherence) without ^15^N decoupling published by Davis *et al.* [18]. The ^15^N resonances of proline and histidine (pH 8.5) were detected by a HA(CA)N pulse sequence according to [19,20], which uses a polarization transfer from ^1^H^α^ proton to backbone ^15^N via ^13^C^α^ and return. In 2D spectra the digital resolution in ^15^N dimension was 0.16 Hz and in ^1^H 0.12 Hz. Proton resonances were additionally measured with the PURGE (Presaturation utilizing relaxation gradients and echoes) sequence [21] with a digital resolution of 0.02 Hz and were referenced to DSS used as internal standard. ^15^N chemical shifts were referenced indirectly to DSS using a ^15^N/^1^H -ratio of 0.101329118 [22].

### 3.5. Data Evaluation

Data processing, spectral analysis and peak picking was performed with BRUKER Topspin 2.1. The pressure dependence of chemical shift δ was fitted to Equation (3) with QtiPlot 0.9.8 using a Scaled Levenberg-Marquardt algorithm.

## 4. Conclusions 

We have presented here random-coil values for both the amide proton and nitrogen that can be used to correct for unspecific pressure effects in proteins. It is also important to show residual structure in disordered polypeptides (see e.g., [23]). The use of a high magnetic field proved to be essential to obtain second order pressure coefficients with sufficient accuracy.
